# Left-Sided Hepatectomy With a Linear Stapling Device: An Experimental Study on Pigs

**DOI:** 10.1155/1992/84680

**Published:** 1992

**Authors:** Thomas Zilling, Bruno S. Walther, Torsten Holmin

**Affiliations:** Department of Surgery Lund University Lund S-221 85 Sweden

## Abstract

Thirteen pigs underwent resection of the left liver lobe. By random selection, the animals were resected either with the aid of an RLG 90^R^ linear stapling device or by the conventional finger-fracture technique. There was one postoperative death due to anaesthetic complications. The median operative time using the stapler was 27 min (range 19–40 min) which was significantly shorter (*p* = 0.0065) than that required for resection by the finger-fracture technique (42.5 min; range 37–55 min).

The median blood loss, estimated by counting the number of gauze swabs used, was 425 ml and 275 ml for the finger-fracture resected and stapler resected groups, respectively (ranges 275–550 ml versus 175– 300 ml; *p* = 0.015).

The animals were sacrificed and examined one week after the operative procedure. Except for a small bile pseudo-cyst in one pig operated upon with conventional resection, no sign of bleeding or biliary leakage was revealed.

This study demonstrates the feasibility of stapling the liver to facilitate resection.


*HPB Surgery*, 1992, Vol. 6, pp. 51–55
Reprints available directly from the publisher
Photocopying permitted by license only

© 1992 Harwood Academic Publishers GmbH
Printed in the United Kingdom

### LEFT-SIDED HEPATECTOMY WITH A LINEAR STAPLING DEVICE: AN EXPERIMENTAL STUDY ON PIGS

THOMAS ZILLING, BRUNO S. WALTHER and TORSTEN HOLMIN
*Department of Surgery, Lund University, Lund, Sweden*

*(Received 9 January 1992)*

Thirteen pigs underwent resection of the left liver lobe. By random selection, the animals were resected either with the aid of an RLG $90^R$ linear stapling device or by the conventional finger-fracture technique. There was one postoperative death due to anaesthetic complications. The median operative time using the stapler was 27 min (range 19–40 min) which was significantly shorter ($p = 0.0065$) than that required for resection by the finger-fracture technique (42.5 min; range 37–55 min).

The median blood loss, estimated by counting the number of gauze swabs used, was 425 ml and 275 ml for the finger-fracture resected and stapler resected groups, respectively (ranges 275–550 ml versus 175–300 ml; $p = 0.015$).

The animals were sacrificed and examined one week after the operative procedure. Except for a small bile pseudo-cyst in one pig operated upon with conventional resection, no sign of bleeding or biliary leakage was revealed.

This study demonstrates the feasibility of stapling the liver to facilitate resection.

KEY WORDS: Left-sided hepatectomy, linear stapling device, pig

### INTRODUCTION

The major problem associated with liver resection is the control of bleeding. Together with infection and liver failure these factors are the major causes of postoperative morbidity and mortality after liver resection[1–3]. Most surgical centres with experience of liver resection report postoperative mortality rates ranging between 3.2 and 14.3%[4–6]. Liver resection in patients with liver trauma is associated with considerably higher (35–70%) mortality rates[7].

In 1908 Pringle described the temporary occlusion of the portal triad[7]. This measure as well as a variety of techniques, such as ultrasonic dissection[8], water jet dissection[9] and resection with the aid of the Nd-YAG laser[10,11], have been designed to limit intraoperative bleeding. Recent reports on the use of linear staplers to control bleeding from the spleen[12,13] have focused on the possibility of using linear stapling devices in the surgery of parenchymatous organs.

In a previous study we have evaluated segmental liver resection with linear stapling devices in pigs. The experiments demonstrated that such operations could be performed quickly and safely[14]. The present study compares stapler technique with the conventional finger-fracture technique in pigs undergoing left-sided hepatectomy.

Address correspondence to: Thomas Zilling M.D., Department of Surgery, Lund University, S-221 85 Lund, Sweden

### MATERIALS AND METHODS

### *Animals*

The pigs were fasted for 24 hours prior to the operative procedure. A total of 21 Swedish domestic pigs were used for the experiments.

Thirteen pigs of random sex underwent liver resection. The median body weights in the stapled and finger-fracture resected groups were 16.5 and 17.3 kg, respectively (ranges 14.8–20.1 and 13.8–18.9 kg).

In a separate series, eight pigs, operated upon for other purposes, underwent total hepatectomy after sacrifice. The livers were divided into their left and right portions and weighed.

### SURGICAL PROCEDURES

The pigs were anaesthetized with Azaperon (R 1929) and Metomidate Hydrochloride (Janssen Pharmaceutica, Beerse, Belgium) and endotracheal intubation performed. The animals were artificially ventilated with a 70%–30% mixture of nitrous oxide and oxygen. During the operation, one litre of Ringer-glucose (Kabi Baxter Infusion AB, Stockholm, Sweden) was given as an infusion.

A laparotomy through a midline incision was performed under sterile conditions. The falciform and triangular ligaments were divided. In the operating room, the pigs were blindly randomized to resection of the left lobe of the liver either with a RLG 90® stapling device (Ethicon Ltd, Somerville, New Jersey, USA) or by the conventional finger-fracture technique. When performing a resection with the stapler, the lateral segment of the left lobe of the liver was introduced into the jaws of the instrument. The instrument was closed and fired and the specimen excised (Figure 1). A few venous or arterial vessels had to be ligated separately. The procedure was repeated for the resection of the medial segment of the left lobe of the liver. The two segments of the left liver lobe in pigs correspond to the left liver lobe in humans.

The time for performing the resection was measured, and the number of gauze swabs used was counted to give an estimation of the blood loss. Seven days postoperatively, arterial blood samples for haemoglobin were taken, the pigs were sacrificed, and an autopsy was performed to inspect the area of resection.

### STATISTICAL METHODS

Difference between groups was determined by using the Mann-Whitney U-test. Correlation between groups was calculated by the Spearman Rank Correlation test.

### RESULTS

The median time for left-sided hepatectomy was significantly shorter in the stapled group as compared to the finger-fracture resected group (27 min versus 42.5 min; ranges 19–40 and 37–55 min, respectively; $p = 0.0065$).

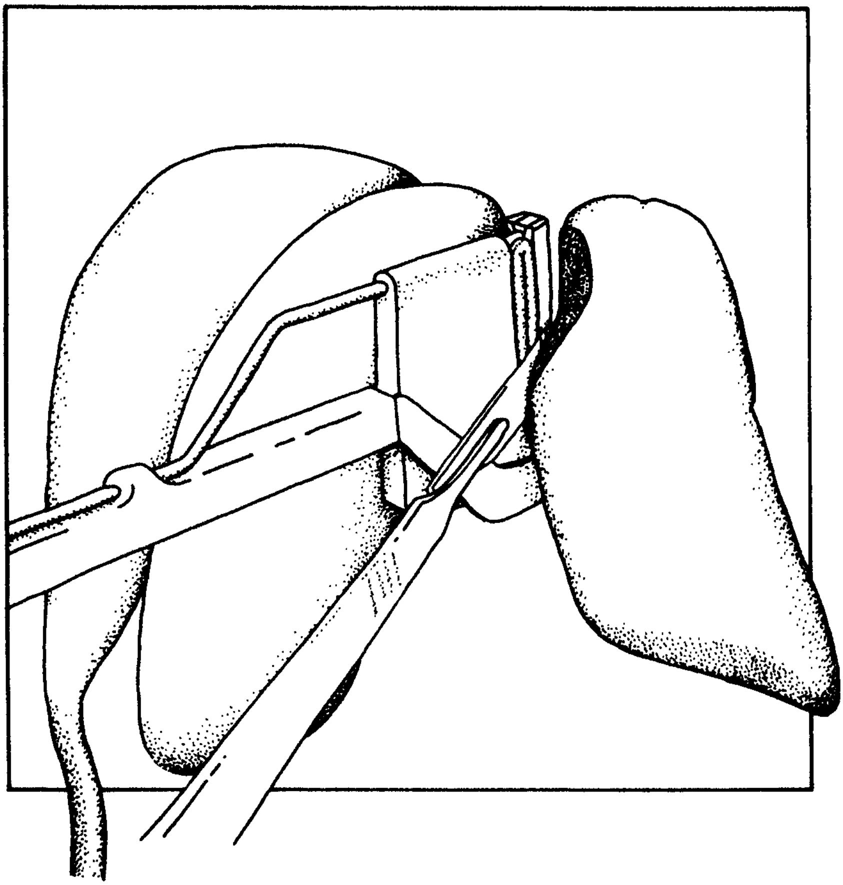

**Figure 1** Left-sided hepatectomy with a RLG 90$^{R}$ linear stapling device. After closure and firing of the instrument, the resected specimen is cut away. The procedure is repeated for the resection of the medial segment of the left liver lobe.

The median postoperative haemoglobin value was lower in the finger-fracture resected group as compared to the stapler resected group (91 versus 101 g/l; ranges 72–101 and 92–104 g/l, respectively). However, there was no significant difference between the groups ($p = 0.054$). The estimated median blood loss by counting the number of compresses was 275 ml for the stapler resected group and 425 ml for the finger-fracture resected group (ranges 175–300 ml and 275–550 ml, respectively; $p = 0.015$). A negative correlation existed between estimated blood loss and postoperative haemoglobin value ($r = -0.737$; $p = 0.003$).

The median weight of the resected liver specimens was 150 g (range 118–186 g) and 159 g (135–201 g) in the stapler resected and finger-fracture resected groups, respectively. The calculated resection size amounted to 26.5 and 30.6% of the total liver weight in the two experimental groups.

At the post-mortem examination one week postoperatively there was a small bile pseudo-cyst in one of the pigs operated upon with finger-fracture technique. No signs of bleeding or biliary leakage were observed in any of the other animals. The weight distribution of the left and right liver lobes of the pigs in the separate series is given in Table 1.

**Table 1** Weight distribution of right and left liver lobes in eight pigs

| *Right (g)* | *Left (g)* | *Weight of the pig (kg)* |
|---|---|---|
| 245 | 321 | 18.0 |
| 260 | 240 | 17.8 |
| 319 | 235 | 19.8 |
| 307 | 308 | 20.5 |
| 367 | 350 | 21.0 |
| 208 | 198 | 16.3 |
| 263 | 252 | 19.0 |
| 287 | 245 | 20.3 |
| 51.3% | 48.7% | |

### DISCUSSION

Many different techniques and devices have been used in an attempt to simplify dissection of liver parenchyma since this procedure carries a substantial risk of injury to major hepatic veins, often resulting in severe bleeding. The ultrasonic dissector and the water jet dissector facilitate parenchymal dissection[8,9]. However, both of these techniques are time consuming and often available only at hospitals with a special interest in liver surgery.

The use of stapling devices in liver surgery seems attractive. The dissection of the parenchyma is performed by the stapler, and near total control of bleeding will be achieved, since most blood vessels (and all bile ducts) will be enclosed by the stapler row.

In a pilot study, five pigs underwent right-sided hepatectomy using a linear stapling device. The procedure was easy to perform but, in the pig the inferior vena cava enters into the right lateral part of the liver and had to be divided. In this first series there were three postoperative deaths. There were no complications from the resection itself, but the complete deviation of blood flow from the inferior vena cava was not well tolerated. In the pig the left and right sides of the liver are almost equal in size (Table 1). We therefore decided to perform left-sided hepatectomy in the present study and found that this procedure was considerably easier and significantly quicker using a stapling device than by the conventional technique.

The findings of a higher (stapled group) median postoperative haemoglobin value and a significantly lower estimated blood loss indicate better control of the bleeding. Voyles and coworkers have recently used linear stapling devices to control intra-operative bleeding from the hepatic veins in liver resections in humans, but they did not perform the dissection of the parenchyma with the stapler[15].

Can staplers simplify liver resections in humans? We have performed one small liver resection on a 75-year old woman with a right-sided liver metastasis from an ovarian cancer. This metastasis was localized peripherally and easy to resect with a linear stapling device. Provided that the liver tissue is not too thick, it should be possible to perform peripheral resections and left lateral segmentectomies in humans. However, it is obvious that major liver resection in man requires further development of the stapling devices.

*Acknowledgements*
Medical Faculty, University of Lund, Lund, Sweden and Johnsson & Johnsson AB, Stockholm, Sweden.

*References*

1. Ekberg, H. (1986) Colorectal liver cancer, resection and regional chemotherapy. Thesis, Lund, Sweden
2. Thompson, H.H., Tompkins, R.K. and Longmire, W.P. (1983) Major hepatic resection. A 25-year experience. *Ann. Surg.*, **197**, 375--388
3. Iwatsuki, S., Shaw, B.W. and Starzl, T.E. (1983) Experience with 150 liver resections. *Ann. Surg.*, **197**, 247-253
4. Logan, S.E., Meier, S.J., Ramming, K.P., Morgon, D.L. and Longmire, W.P. (1982) Hepatic resection of metastatic colo-rectal carcinoma -- A ten years experience. *Arch. Surg.*, **117**, 25-28
5. Li, G.H., Zhus, S.L., Li, J.Q. and Zhan, Y.Q. (1989) Evaluation of partial hepatectomy for primary liver carcinoma. *J. Surg. Oncol.*, **41**, 5-8
6. Iwatsuki, S. and Starzl, T.E. (1988) Personal experience with 411 hepatic resections. *Ann. Surg.*, **208**, 421-434
7. Walt, A.J. and Bender, J.S. (1985) *Injuries of the liver from Maingot's Abdominal Operations*. 8th ed., pp. 1577-1590, ISBN 0-8385-6099-7
8. Hodgson, W.J. and DelGuecio, L.R. (1984) Preliminary experience in liver surgery by using the ultrasonic scalpel. *Surgery*, **95**, 230-234
9. Persson, B.G., Jeppsson, B., Tranberg, K.G., Roslund, K. and Bengmark, S. (1989) Transection of the liver with a water jet. *Surg. Gynecol. Obstet.*, **168**, 267-268
10. Joffe, S.N., Brackett, K.A., Sankar, M.Y. and Daikuzono, N. (1986) Resection of the liver with the Nd:YAG laser. *Surg. Gynecol. Obstet.*, **163**, 437-442
11. Tranberg, K.G., Rigotti, P., Brackett, K.A., Björnson, H.S., Fischer, J.E. and Joffe, S.N. (1986) Liver resection. A comparison using the Nd-YAG laser, an ultrasonic surgical aspirator, or blunt dissection. *Am. J. Surg.*, **151**, 368-373
12. Breil, Ph. and Bahnini, M.A., Fékété, F. (1989) Par5tial splenectomy using the TA stapler. *Surg. Gynecol. Obstet.* **163**, 575-576
13. Raschbaum, G., Harnar, T. and Canizaro, P. (1988) The use of a stapler in splenic salvage as an alternative to the sutured partial splenectomy or splenography. *Surg. Gynecol. Obstet.*, **166**, 179--180
14. Zilling, T., Walther, B.S. and Holmin, T. (1990) Segmental liver resection with linear stapling devices. *In vivo*, **4**, 273-276
15. Voyles, C.R., Vogel, S.B. (1989) Hepatic resection using stapling devices to control the hepatic veins. *Am. J. Surg.*, **158**, 459-460

(*Accepted by S. Bengmark 13 January 1992*)

